# Chronological and Skeletal Age in Relation to Physical Fitness Performance in Preschool Children

**DOI:** 10.3389/fped.2021.641353

**Published:** 2021-05-14

**Authors:** Dandan Ke, Dajiang Lu, Guang Cai, Xiaofei Wang, Jing Zhang, Koya Suzuki

**Affiliations:** ^1^Graduate School of Health and Sports Science, Juntendo University, Inzai, Japan; ^2^School of Kinesiology, Shanghai University of Sport, Shanghai, China; ^3^Shanghai Research Institute of Sports Science, Shanghai, China; ^4^Shanghai Center for Women and Children's Health, Shanghai, China

**Keywords:** physical activity, motor performance, motor competence, bone age, growth and maturation, pediatrics

## Abstract

**Introduction:** Physical fitness is an adaptive state that varies with an individual's growth and maturity status. Considering that the difference in skeletal maturity already existed among preschool children, this study was designed to determine the influence of skeletal age and chronological age on preschoolers' physical fitness performance.

**Methods:** This cross-sectional study was conducted in 945 healthy preschoolers (509 males, 436 females) aged between 3.0 and 6.0 years in Shanghai, China. We used the method of TW3-C RUS to determine skeletal age. Chronological age was measured by subtracting the date of birth from the test date. Sit and reach, 2 × 10 m shuttle run test, standing long jump, tennis ball throw, 5 m jump on both feet, and balance beam walk were considered for physical fitness performance. Correlation coefficients and partial correlations adjusting height and weight were used to determine the relationships among the variables of skeletal age/ relative skeletal age, chronological age/relative chronological age, and physical fitness items.

**Results:** Skill-related physical fitness was weakly to moderately associated with skeletal age (the absolute value of r: 0.225–0.508, *p* < 0.01) and was moderately to strongly associated with chronological age (the absolute value of r: 0.405–0.659, *p* < 0.01). Health-related physical fitness items (BMI and sit and reach) showed a fairly weak to no correlation with skeletal age and chronological age. After adjusting the height and weight, an extremely weak to no correlation was observed between skeletal age and both health- and skill-related physical fitness, and weak-moderate correlations were noted between chronological age and skill-related physical fitness (the absolute value of r: 0.220–0.419, *p* < 0.01). In children in Grade 1, skill-related physical fitness (except for balance beam walk) showed a weak to moderate correlation with relative chronological age (the absolute value of r: 0.227–0.464, *p* < 0.05).

**Conclusion:** (1) both skeletal age and chronological age are associated with skill-related rather than health-related physical fitness performance, and after adjusting height and weight, chronological age, rather than skeletal age, is associated with skill-related physical fitness performance; (2) for preschool children, skill-related physical fitness performance is influenced by relative chronological age rather than individual differences in skeletal maturation, especially in the lower grades.

## Introduction

Physical fitness refers to the ability of the body systems to work effectively in harmony to enjoy leisure time, stay healthy, and cope with emergencies (1). Monitoring of physical fitness in children should receive considerable critical attention because it has previously been observed that physical fitness is not only positively associated with academic achievement and cognitive functions (2–4) but also is a potent health marker of cardiovascular, metabolic, and skeletal health (5) in childhood and adolescence. Further, a longitudinal study showed a moderate to a highly significant correlation between health-related physical fitness components in childhood and those in adulthood (6). With the growing public health concern worldwide, there is an overall declining trend of physical fitness among children (7). Hence, the improvement and maintenance of physical fitness have become the main challenges for many researchers.

Physical fitness is an adaptive state relevant to the individual's growth and maturity status, lifestyle, and environmental factors (1, 8). The preschool age is a critical period for dramatic physiological changes, neuromuscular development, and the acquisition of fundamental motor skills (8). Thus, the influencing factors of physical performance in early childhood are more complicated. Several attempts have been made to examine the effects of lifestyle (i.e., physical activity, nutrition intake, and sedentary behaviors) (9, 10) and environment (i.e., kindergarten, family, and community) (11, 12) on preschooler's physical fitness performance. Growth and maturity are suggested to be the most important factors influencing young children's physical fitness (8); however, there is much less evidence on this.

An individual's growth and maturity are usually determined by chronological age and biological age. The chronological age is easily determined by the date of birth, and it has been used as the age category in the physical fitness standards worldwide. As mentioned in extensive research, chronological age significantly influences physical fitness in preschool children (13, 14). In recent years, there has similarly been an increasing interest in the impact of relative chronological age (the difference in birth month) on the physical performance of preschool children of the same grade (15). The biological age is usually expressed by the skeletal age, which reflects the actual physical growth and maturity status. Previous studies on adolescents have reported that the skeletal maturation status significantly affects their physical performance (16–18), and maturity-age should be used during the selection and competition of young athletes (19, 20). Relevant studies on preschool children are extremely limited, and there is only one study conducted among children aged 3–6 years demonstrating that skeletal maturation has a relatively minimal effect on fundamental motor skills and motor performance (21). However, as shown in our previous study, the difference in skeletal age at the same chronological age is as high as 2.0 years (22). This could result in a significant difference in height and weight (23) that might steeply influence physical fitness performance. However, there are no published data on the association between skeletal age and physical fitness among preschool children, especially after adjusting height and weight.

Therefore, a cross-sectional study was designed among a cohort of Chinese preschool children to determine the influence of skeletal age and chronological age on their physical fitness performance. This research could contribute to providing some evidence on the importance of the effect of skeletal age and chronological age on the physical fitness of preschool children, which might be useful for physical education practitioners who are concerned about targeted methods to improve preschooler's physical fitness levels.

## Materials and Methods

This study was approved by the Shanghai Nutrition Society Medical Ethics Committee (No. 2019–007) on July 11, 2019, and the participants' confidentiality was strictly maintained throughout the study. Prior to participation, the purpose and procedures of this study were explained to the participants' parents and teachers in each kindergarten. In addition, written informed consent was obtained from the parents.

### Participants

**Figure 1:**
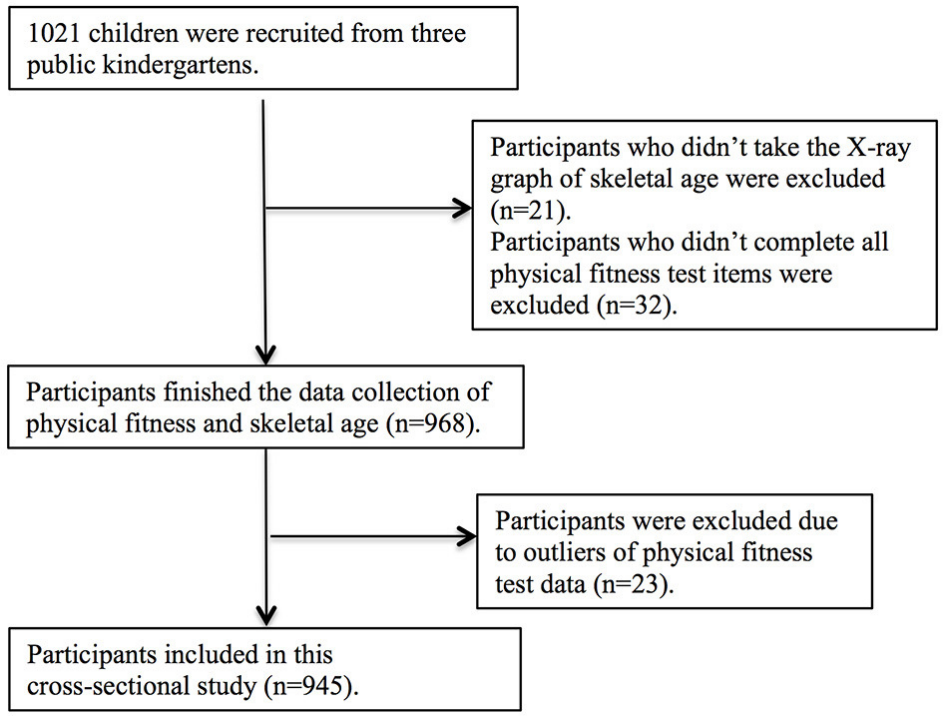
Flowchart of sample selection process.

This cross-sectional study was conducted at school entry (autumn 2019) in Shanghai, China. The participants were recruited from three non-randomly selected public kindergartens rated as First-level Kindergarten (24), and notably, all children were recruited, except for those in four classes who were quarantined due to influenza. Written study information and informed consent forms were sent to the participants' guardians. In addition, the teachers of the kindergartens were informed about the study introduction content. The participants were formally included in the study after the informed consent was received. In total, 1021 Chinese children were registered for this study. After excluding the missing data and outliers rejected, a total of 945 participants (509 males, 4.8 ± 0.8 years; 436 females, 4.8 ± 0.8 years) were included in the final analyses. The sample selection process is detailed in Figure 1.

### Chronological and Skeletal Age

The individual chronological age was measured by subtracting the calendar birth date from the test date. Since the enrollment age in China was calculated as ending on August 31, relative chronological age was calculated by dividing the difference of birth date and August 31 by 365, and the difference is specifically the gap between the date of birth and August 31 (for children born from January to August) or August 31 of the following year (for children born from September to December).

The determination of skeletal age has been previously reported (22). Briefly, the skeletal age was assessed by comparing the obtained X-ray film of the left hand and wrist, which was taken with a digital portable X-ray apparatus (MOVIX4.0+D Ream, Stephanie, France) with the standard of Tanner-Whitehouse 3-Chinese Radius-Ulna-Short Bones (TW3-C RUS) (25). The reliability of skeletal age evaluation has been reported in our previous study (22). The relative skeletal age is determined by the difference between the skeletal age and the chronological age (skeletal age minus chronological age), which indicates skeletal maturity (8).

### Physical Fitness Assessment

The participant' weight (0.1 kg) and height (0.1 cm) were measured by a mechanical stadiometer (Ningbo Finer Medical Instruments Co., Limited, Zhejiang, China) and a body fat and weight measurement device (V-BODY HBF-371, Omron, Japan), respectively, without shoes and coats on. Body mass index (BMI) was calculated using the equation as follows: BMI (kg/m^2^) = Weight (kg)/Height^2^ (m^2^). The physical fitness performance was determined according to the Chinese National Physical Fitness Measurement (preschool children version) (26). The test battery is widely used in China as an official manual, and a previous study reported its reliability and validity (27). This physical fitness test battery includes six test items: sit and reach, 2 × 10 m shuttle run test, standing long jump, tennis ball throw, 5 m jump on both feet, and balance beam walk. The test was conducted by trained researchers in the activity room and playground of each kindergarten. The assessment details are as follows.

#### Sit and Reach

The participants removed their shoes and were instructed to sit on the floor with legs fully extended with feet against the front end of the test equipment (Reliable Co., Ltd., Beijing, China), and then slowly bend forward to push the Vernier as far as possible with their fingertips. The participants performed the test twice, and the better result was recorded (0.1 cm). Longer distance reflects better flexibility.

#### 2 × 10 m Shuttle Run

The test was conducted between groups of two participants. A marker tube was placed on the turn-back line of the 10 m long and 1.22 m wide runways, respectively. The participants placed their front foot behind the starting line. With the signal “Go!,” the participants sprinted to the turn-back line, touched the tube, and ran back across the finish line. The participants performed the test once and recorded the time spent (0.1 s). The shorter time spent reflects better speed of movement and agility.

#### Standing Long Jump

The participants jumped as far as possible with their feet together (separate from each other at about the shoulder's width) while staying upright. The distance between the start line and the landing position was measured. The participants performed this test twice, and the better result was recorded (1.0 cm). Longer distance reflects greater lower limb explosive strength.

#### Tennis Ball Throw

The participants stood in front of the starting line and were instructed to hold one tennis ball with their dominant hands and throw (overhand) it as far as possible. The longer distance of the two attempts was recorded as the test score (0.5 m), and longer distance reflects greater upper limb power/strength and better coordination.

#### 5 m Jump on Both Feet

Ten horizontal lines were drawn 0.5 m apart on the flat ground, and a soft square bag (length 10 cm, width 5 cm, height 5 cm) was placed on each line. The participants stood in front of the start line, and upon the signal “start,” continuously skipped 10 bags. The observer recorded the time spent (the better of the two attempts) as the test result (0.1 s). The shorter time spent reflects better coordination and lower limb strength.

#### Balance Beam Walk

The participants walked over the balance beam (3 m long, 10 cm wide, and 30-cm high; a square platform with the same height and a side length of 20 cm was added to both ends of the balance beam as the starting and ending areas) (Reliable Co., Ltd., Beijing, China) at the fastest speed without falling down. Each participant walked twice, and the lesser time required was recorded as the result (0.1 s). The shorter time spent reflects better dynamic balance.

### Statistical Analyses

The data were entered into an Excel spreadsheet and imported into SPSS Statistics for Mac. Version 21.0 (IBM Co., Armonk, NY, USA) for analyses. All variables were expressed as the mean ± standard deviation. The differences in each measurement parameter between the chronological age and skeletal age groups were analyzed using one-way analysis of variance (ANOVA), and differences across genders were analyzed using analysis of the independent-samples *t*-test. Subsequently, a Spearman's correlation analysis was performed between chronological age, skeletal age, body size (height and weight), and physical fitness tests in gender groups. The relationship between relative chronological age, relative skeletal age, body size (height and weight), and physical fitness was evaluated using Pearson correlation coefficient in gender and grade groups. Additionally, partial correlations, adjusting height and weight, were used to determine the relationships among the variables of skeletal age, chronological age, relative skeletal age, relative chronological age, and physical fitness items. Notably, the differences were considered statistically significant at *p* < 0.05.

## Results

The means and standard deviations by chronological age group and gender are stated in Table 1, and in addition, analyses by skeletal age group are presented in Supplementary Table 1. As shown in Table 1, from the 3.0-year group to the 6.0-year group, male's height increased from 99.2 ± 3.9 to 119.1 ± 5.9 cm, and weight increased from 16.2 ± 2.0 to 24.0 ± 3.6 kg. Similarly, female's height increased from 97.9 ± 3.3 to 118.1 ± 4.7 cm, and weight increased from 15.6 ± 1.9 to 22.5 ± 3.4 kg, and significant differences were observed between age groups. Regarding health-related physical fitness, there was no significant change in BMI and female's sit and reach, except for male's sit and reach performance, which decreased from 10.9 ± 3.6 cm in the 3.0-year group to 5.5 ± 4.7 cm in the 6.0-year group (*p* < 0.01). Skill-related physical fitness performance significantly improved with age (*p* < 0.01). Regarding sit and reach that reflects flexibility, the females consistently performed better than males in five of the seven age groups (*p* < 0.01); on the contrary, in four of the seven age groups, males significantly showed better performance than females in tennis ball throw (*p* < 0.05). In other items, gender-related differences were relatively small, and a similar result is presented in Supplementary Table 1 grouped by skeletal age.

**Table 1:** Descriptive statistics for body size, physical fitness by gender and chronological age.

	**N**	**Body size**		**Health-related physical fitness**		**Skill-related physical fitness**				
		**Height (cm)**	**Weight (kg)**	**BMI (kg/m^**2**^)**	**Sit and reach (cm)**	**2 × 10 m SRT (s)**	**Standing long jump (cm)**	**Tennis ball throw (m)**	**5 m jump on both feet (s)**	**Balance beam walk (s)**
**Male (*****n*** **= 509)**										
3.0 ~ <3.5	36	99.2 ± 3.9	16.2 ± 2.0	16.4 ± 1.1	10.9 ± 3.6	12.5 ± 2.8	50.0 ± 17.1	2.4 ± 1.1	10.7 ± 4.6	29.9 ± 16.0
3.5 ~ <4.0	66	101.7 ± 4.3	17.1 ± 2.3	16.5 ± 1.5	9.8 ± 4.2	11.0 ± 2.3	59.6 ± 18.2	3.0 ± 1.0	9.0 ± 4.0	26.4 ± 14.2
4.0 ~ <4.5	59	106.7 ± 4.6	18.9 ± 2.7	16.5 ± 1.8	9.4 ± 4.3	9.5 ± 1.6	73.3 ± 15.4	3.6 ± 1.3	7.6 ± 2.8	22.0 ± 11.7
4.5 ~ <5.0	106	109.2 ± 4.2	19.1 ± 2.2	16.0 ± 1.3	8.7 ± 4.7	9.0 ± 1.5	82.5 ± 17.7	3.9 ± 1.4	7.7 ± 3.2	20.0 ± 14.7
5.0 ~ <5.5	104	112.4 ± 4.4	20.5 ± 3.1	16.1 ± 1.9	7.0 ± 4.4	8.2 ± 1.0	87.9 ± 17.9	4.7 ± 1.7	6.1 ± 2.0	15.7 ± 11.5
5.5 ~ <6.0	107	115.9 ± 4.8	22.0 ± 4.0	16.3 ± 2.2	8.0 ± 5.2	7.9 ± 1.1	95.5 ± 15.5	5.6 ± 1.8	5.7 ± 1.8	11.1 ± 10.2
6.0 ~ <6.5	31	119.1 ± 5.9	24.0 ± 3.6	16.8 ± 1.7	5.5 ± 4.7	7.9 ± 1.2	100.5 ± 17.4	5.7 ± 2.0	5.3 ± 1.0	11.4 ± 7.2
One-way ANOVA		^**^	^**^	ns	^**^	^**^	^**^	^**^	^**^	^**^
**Female (*****n*** **= 436)**										
3.0 ~ <3.5	35	97.9 ± 3.3	15.6 ± 1.9	16.3 ± 1.4	11.8 ± 2.1	12.1 ± 2.3	44.9 ± 17.8	2.1 ± 0.5	9.7 ± 4.2	25.3 ± 14.5
3.5 ~ <4.0	55	101.1 ± 3.6	16.3 ± 1.8^#^	15.9 ± 1.4^#^	11.7 ± 3.3^##^	10.9 ± 1.5	58.9 ± 13.9	2.7 ± 0.8	8.1 ± 2.8	24.2 ± 13.6
4.0 ~ <4.5	58	104.9 ± 4.6^#^	17.0 ± 2.1^##^	15.4 ± 1.1^##^	10.2 ± 3.5	9.8 ± 1.5	70.0 ± 13.0	3.1 ± 1.0^#^	7.3 ± 1.8	24.4 ± 13.7
4.5 ~ <5.0	81	108.3 ± 4.1	18.4 ± 2.4	15.7 ± 1.5	11.9 ± 3.5^##^	9.0 ± 1.4	78.3 ± 14.6	3.5 ± 1.0^#^	6.6 ± 1.6^##^	21.3 ± 13.5
5.0 ~ <5.5	109	112.1 ± 4.6	20.0 ± 2.8	15.9 ± 1.8	11.3 ± 5.1^##^	8.5 ± 1.1^#^	84.3 ± 13.1	4.3 ± 1.3^#^	6.5 ± 2.3	13.7 ± 11.5
5.5 ~ <6.0	73	115.4 ± 5.0	21.4 ± 3.6	16.0 ± 2.2	11.5 ± 4.8^##^	8.4 ± 0.9^##^	87.6 ± 16.5^##^	4.6 ± 1.3^##^	6.1 ± 1.8	13.0 ± 10.2
6.0 ~ <6.5	25	118.1 ± 4.7	22.5 ± 3.4	16.2 ± 3.2	12.7 ± 3.7^##^	8.1 ± 0.7	93.6 ± 12.3	5.4 ± 1.5	5.7 ± 1.2	10.3 ± 9.1
One-way ANOVA		^**^	^**^	ns	ns	^**^	^**^	^**^	^**^	^**^

** *p < 0.01*.

# *Female vs. Male, p < 0.05*,

## *Female vs. Male, p < 0.01*.

*BMI, body mass index; SRT, shuttle run test; ns, no significance*.

The relationships between chronological age, skeletal age, height, weight, and physical fitness are presented in Table 2. This table includes the coefficients of correlations and partial correlations (adjusting height and weight) between ages and physical fitness items. As shown, height and weight are strongly associated with skeletal age and chronological age (*p* < 0.01), respectively. Skill-related physical fitness performance was weakly-moderately associated with skeletal age (the absolute value of r: 0.225–0.508, *p* < 0.01) and moderately-strongly associated with chronological age (the absolute value of r: 0.405–0.659, *p* < 0.01). However, health-related physical fitness items (BMI and sit and reach) showed a fairly weak to no correlation with skeletal age and chronological age, respectively. After adjusting the height and weight, there was no or very weak correlation between skeletal age and both health- and skill-related physical fitness. In contrast, weak-moderate correlations were observed between chronological age and skill-related physical fitness (the absolute value of r: 0.220–0.419, *p* < 0.01).

**Table 2:** Correlations and partial correlations between skeletal age, chronological age, and body size, physical fitness in children aged 3–6 years old.

	**SA**		**CA**		**Height (cm)**	**Weight (kg)**	**BMI (kg/m^**2**^)**
	**r**	**r_**partial-H&W**_**	**r**	**r_**partial-H&W**_**			
**Male (*****n*** **=** **509)**							
Height (cm)	0.735^**^	–	0.777^**^	–	–	–	–
Weight (kg)	0.629^**^	–	0.621^**^	–	0.778^**^	–	–
BMI (kg/m^2^)	0.096^*^	0.078	−0.046	0.112^*^	0.069	0.672^**^	–
Sit and reach (cm)	−0.0193^**^	0.012	−0.244^**^	−0.063	−0.259^**^	−0.138^**^	0.084
2 × 10 m SRT (s)	−0.427^**^	−0.062	−0.640^**^	−0.419^**^	−0.514^**^	−0.341^**^	0.047
Standing long jump (cm)	0.508^**^	0.137^**^	0.641^**^	0.405^**^	0.553^**^	0.363^**^	−0.057
Tennis ball throw (m)	0.382^**^	−0.006	0.580^**^	0.287^**^	0.524^**^	0.411^**^	0.044
5 m jump on both feet (s)	−0.306^**^	−0.033	−0.513^**^	−0.306^**^	−0.368^**^	−0.288^**^	−0.035
Balance beam walk (s)	−0.266^**^	−0.039	−0.479^**^	−0.303^**^	−0.319^**^	−0.209^**^	0.034
**Female (*****n*** **=** **436)**							
Height (cm)	0.668^**^	–	0.801^**^	–	–	–	–
Weight (kg)	0.606^**^	–	0.652^**^	–	0.764^**^	–	–
BMI (kg/m^2^)	0.153^*^	0.04	0.016	0.129^*^	0.014	0.651^**^	–
Sit and reach (cm)	−0.013	−0.065	0.043	0.002	0.026	0.05	0.050
2 × 10 m SRT (s)	−0.486^**^	−0.118^*^	−0.622^**^	−0.348^**^	−0.565^**^	−0.419^**^	0.002
Standing long jump (cm)	0.458^**^	0.125^**^	0.645^**^	0.416^**^	0.576^**^	0.396^**^	−0.047
Tennis ball throw (m)	0.428^**^	−0.011	0.659^**^	0.345^**^	0.579^**^	0.457^**^	0.031
5 m jump on both feet (s)	−0.225^**^	0.000	−0.405^**^	−0.232^**^	−0.305^**^	−0.239^**^	−0.010
Balance beam walk (s)	−0.269^**^	−0.027	−0.467^**^	−0.220^**^	−0.310^**^	−0.248^**^	−0.013

*CA, chronological age; SA, skeletal age; BMI, body mass index; SRT, shuttle run test; partial-H&W, partial-Height & Weight*.

* *p < 0.05*,

** *p < 0.01*.

Table 3 shows the correlations and partial correlations of relative chronological age and relative skeletal age with height, weight, and physical fitness. Height and weight showed a weak to moderate correlation with relative skeletal age in all grade and gender groups (the absolute value of r: 0.219–0.451, *p* < 0.01), and physical fitness items showed very weak or no correlations and partial correlations with relative skeletal age. Regarding relative chronological age, in Grade 1, skill-related physical fitness items (except for balance beam walk) showed weak-moderate correlation with relative chronological age (the absolute value of r: 0.227–0.464, *p* < 0.05), and after adjusting height and weight, most test items still showed weak correlations. However, in Grade 2 and 3, the items and correlation coefficients associated with the relative chronological age decreased, and in Grade 3, only standing long jump showed a very weak correlation with relative chronological age (male, *r* = 0.210, *p* < 0.01, female, *r* = 0.157, *p* < 0.05).

**Table 3:** Correlations and partial correlations between relative skeletal age, relative chronological age, and body size, physical fitness in children aged 3–6 years old.

	**RSA**		**RCA**		**RSA**		**RCA**	
	**r**	**r_**partial-H&W**_**	**r**	**r_**partial-H&W**_**	**r**	**r_**partial-H&W**_**	**r**	**r_**partial-H&W**_**
**Grade 1**	**Male (*****n*** **=** **118)**				**Female (*****n*** **=** **108)**			
Height (cm)	0.333^**^	–	0.492^**^	–	0.219^*^	–	0.449^**^	–
Weight (kg)	0.334^**^	–	0.344^**^	–	0.281^**^	–	0.175	–
BMI (kg/m^2^)	0.181	0.049	0.049	0.066	0.175	0.041	−0.177	−0.075
Sit and reach (cm)	0.035	0.05	−0.189^*^	−0.194^*^	0.219^*^	0.165	−0.160	−0.216^*^
2 × 10 m SRT (s)	0.094	0.155	−0.343^**^	−0.302^**^	0.020	0.091	−0.324^**^	−0.246^*^
Standing long jump (cm)	−0.133	−0.225^*^	0.358^**^	0.287^**^	0.042	−0.051	0.464^**^	0.362^**^
Tennis ball throw (m)	0.012	−0.102	0.365^**^	0.271^**^	−0.173	−0.254^**^	0.433^**^	0.383^**^
5 m jump on both feet (s)	0.044	0.106	−0.227^*^	−0.186^*^	−0.052	−0.040	−0.237^*^	−0.194^*^
Balance beam walk (s)	0.036	0.093	−0.187^*^	−0.138	0.104	0.051	−0.143	−0.149
**Grade 2**	**Male (*****n*** **= 186)**				**Female (*****n*** **= 167)**			
Height (cm)	0.367^**^	–	0.246^**^	–	0.227^**^	–	0.451^**^	–
Weight (kg)	0.360^**^	–	0.013	–	0.237^**^	–	0.297^**^	–
BMI (kg/m^2^)	0.169^*^	0.009	−.0207^**^	0.055	0.133	0.002	0.020	0.112
Sit and reach (cm)	−0.042	−0.010	−0.153^*^	−0.096	−0.064	−0.068	0.095	0.120
2 × 10 m SRT (s)	0.012	0.041	−0.210^**^	−0.154^*^	−0.047	−0.030	−0.142	−0.107
Standing long jump (cm)	0.205^**^	0.159^*^	0.265^**^	0.186^*^	−0.034	−0.078	0.265^**^	0.213^**^
Tennis ball throw (m)	−0.030	−0.087	0.180^*^	0.139	−0.085	−0.155^*^	0.285^**^	0.199^**^
5 m jump on both feet (s)	0.003	0.014	−0.111	−0.091	0.087	0.094	−0.091	−0.100
Balance beam walk (s)	−0.007	−0.024	−0.226^**^	−0.216^**^	0.041	0.080	−0.281^**^	−0.262^**^
**Grade 3**	**Male (*****n*** **=** **205)**				**Female (*****n*** **=** **161)**			
Height (cm)	0.451^**^	–	0.389^**^	–	0.251^**^	–	0.262^**^	–
Weight (kg)	0.355^**^	–	0.252^**^	–	0.331^**^	–	0.171^*^	–
BMI (kg/m^2^)	0.138^*^	0.060	0.054	0.088	0.218^**^	0.011	0.039	0.148
Sit and reach (cm)	−0.008	0.064	−0.011	0.058	−0.109	−0.126	0.068	0.063
2 × 10 m SRT (s)	0.050	0.063	0.006	0.010	−0.107	−0.057	−0.009	0.048
Standing long jump (cm)	0.015	−0.03	0.210^**^	0.188^**^	0.006	0.029	0.157^*^	0.154
Tennis ball throw (m)	0.050	−0.068	0.085	−0.008	−0.061	−0.135	0.170^*^	0.115
5 m jump on both feet (s)	0.066	0.061	−0.030	−0.055	0.156^*^	0.199^*^	−0.028	−0.014
Balance beam walk (s)	0.121	0.133	−0.135	−0.147^*^	0.022	0.073	0.044	0.090

*RSA, relative skeletal age; RCA, relative chronological age; BMI, body mass index; SRT, shuttle run test; partial-H&W, partial-Height & Weight*.

* *p < 0.05*,

** *p < 0.01*.

## Discussion

The main purpose of this study was to demonstrate the associations between skeletal age/relative skeletal age and chronological age/relative chronological age and physical fitness performance in preschool children, especially independent of height and weight. Overall, the main finding was that skill-related physical fitness correlated with relative chronological age rather than relative skeletal age, contrary to the findings of previous studies that suggested that the skeletal maturation status has a much stronger influence on motor performance than the birth quarter and should be used in the selection and competition of young athletes (19, 20, 28). Additionally, both skeletal age and chronological age are associated with skill-related rather than health-related physical fitness performance, and after adjusting the height and weight, the chronological age was weakly-moderately correlated with skill-related physical fitness and skeletal age was not or very weakly correlated with skill-related physical fitness. This result might provide evidence to support the speculation from previous studies that skeletal age influences physical fitness performance mainly through height and weight (29). There are several possible explanations for the results obtained in this study that we try to discuss from three perspectives.

Firstly, the characteristic of the physical fitness item may affect the relationships between chronological age, skeletal age, and physical fitness performance. The physical fitness test includes a series of standardized motor tasks for children (14, 27), and it is more complex than specific motor acts executed in motor competence measuring. An early study (30) showed that correlations between skeletal age and isometric strength measured in the cable tension method are higher than those between skeletal age and motor performance. In this study, two health-related indicators (BMI reflecting body composition and sit and reach reflecting flexibility), reflecting a single physical ability and a simple test method, showed very weak or no correlation with skeletal and chronological age and relative age. However, other skill-related physical fitness test items were weakly-moderately associated with skeletal age and moderately-strongly associated with chronological age (as shown in Table 2), which is consistent with the finding of a previous study (29) that indicated a greater effect of skeletal age on motor fitness than handgrip performance (health-related fitness). Skill-related physical fitness measures more than one component, such as the motor components of coordination and lower limb strength that are assessed in the 5 m jump on both feet test. The early childhood stage is a crucial period for the development of basic motor skills, and significant changes might occur in a short time during this stage (8), which might explain the existence of a relationship between age and skill-related physical fitness performance. Additionally, physical fitness is related to intellectual maturity in preschool children (31), and the complexity of the test method might affect the understanding and mastery of various test methods, which may affect the demonstration of actual competence. This speculation is supported by the findings of previous studies that moderate-to-strong positive correlations exist between actual motor competence and physical fitness in preschool children (32, 33).

Secondly, physical fitness performance is not only decided by growth and maturation but also by adaptation, which is a form of motor learning in virtually all movements (34). Most of the young children are not specifically trained, and the experience of daily learning is crucial to improve motor skills. Consistent with this view, the importance of participation has similarly been highlighted in previous studies, indicating that an increased physical activity contributes to an improved physical fitness performance (32, 35, 36). Additionally, the effect of relative chronological age can be explained from the same perspective. A previous study emphasized the impact of relative chronological age (the difference in birth month) on the physical fitness performance of preschool children (15), and similar results were also obtained in our study (Table 3). Thus, we can speculate that chronological age not only reflects growth and maturation but also implies survival duration in the society, which indicates the opportunity to improve adaptation through participation and experience. On the contrary, skeletal age mainly reflects the growth and maturation status. However, this situation might be gradually altered with an increased time of living and studying in a similar environment. This is because, as shown in Table 3, the effect of relative chronological age seemed to be greater in grade 1 than in grade 3. In addition, previous studies have shown that there are similar correlation coefficients between skeletal age, chronological age and physical fitness in adolescent males (8, 30, 37).

Thirdly, the skeletal maturity status might determine the entire extent of motor skills, such as youth athlete's achievement in a competition after intensive training; however, it does not directly determine the performance of motor tasks in children and adolescents without practice. Hence, skeletal age is used more in competitive sports. It has been suggested that skeletal maturation influences competition performance, which strengthens the importance of skeletal age in selecting young athletes (19, 20, 38). Consequently, much attention is not given to general physical education.

There are a few limitations to this study. Several factors influence physical fitness performance in preschool children; however, this study failed to consider other main influencing factors, physical activity lifestyles, and environmental factors (1) and only comparatively analyzed the effects of chronological age and skeletal age on physical fitness. Hence, we could not quantitatively explain the impact of each factor on physical fitness, and, therefore, could not provide direct and strong evidence for formulating measures to improve children's physical fitness. Secondly, although some test items included muscular strength components, the direct measurement of strength (e.g., handgrip, knee extension, and muscle mass) was not considered. This led to the failure to prove whether the relationship between preschool children's muscle strength and skeletal age and chronological age is different from that among other physical fitness performance. However, there are relatively few general methods for direct measurement of muscle mass and muscle strength in preschool children.

Nevertheless, this study has several strengths. To the best of our knowledge, this is the first study that clarified the correlation coefficients between skeletal age/relative skeletal age, chronological age/relative chronological age and physical fitness, especially adjusting the height and weight. The evidence from this study suggests that (1) both skeletal age and chronological age are associated with skill-related rather than health-related physical fitness performance, and after adjusting height and weight, chronological age, rather than skeletal age, is associated with skill-related physical fitness performance; (2) for preschool children, skill-related physical fitness performance is influenced by relative chronological age rather than individual differences in skeletal maturation, especially in the lower grades.

## Data Availability Statement

The raw data supporting the conclusions of this article will be made available by the authors, without undue reservation.

## Ethics Statement

The studies involving human participants were reviewed and approved by The Shanghai Nutrition Society Medical Ethics Committee (No. 2019–007). Written informed consent to participate in this study was provided by the participants' legal guardian/next of kin.

## Author Contributions

DK and DL designed this study. DK, JZ, GC, and XW carried out the experiments. DK and KS analyzed experimental data. DK was responsible for manuscript writing. KS revised the manuscript. All authors were involved in writing the paper, and had final approval of the submitted and published versions.

## Conflict of Interest

The authors declare that the research was conducted in the absence of any commercial or financial relationships that could be construed as a potential conflict of interest.
