# Supplementary material for: Chronological and Skeletal Age in Relation to Physical Fitness Performance in Preschool Children
Source: Front Pediatr. 2021 May 14;9:641353. doi: 10.3389/fped.2021.641353 (PMC8160222; doi:10.3389/fped.2021.641353)
Supplement: Supplementary file 1 [file Table_1.pdf]

SUPPLEMENTARY TABLE 1. Descriptive statistics for body size, physical fitness by gender and skeletal age

	N	Body size		Health-related physical fitness		Skill-related physical fitness				
		Height (cm)	Weight (kg)	BMI (kg/m^2)	Sit and reach (cm)	2×10 m SRT (s)	Standing long jump (cm)	Tennis ball throw (m)	5 m jump on both feet (s)	Balance beam walk (s)
**Male (n =509)**										
~ < 3.0	42	99.8±4.8	16.1±1.7	16.1±1.1	10.5±4.1	11.0±2.2	58.5±16.6	2.8±1.3	9.4±4.3	26.0±15.7
3.0 ~ < 3.5	44	102.6±4.0	17.2±1.8	16.3±1.4	9.4±3.9	10.6±2.2	62.2±16.9	3.5±1.2	8.2±3.1	24.7±13.6
3.5 ~ < 4.0	55	105.6±5.4	18.3±2.6	16.4±1.6	8.3±4.1	9.8±2.4	73.1±23.5	3.7±1.6	7.9±4.2	24.1±16.4
4.0 ~ < 4.5	88	108.2±5.2	18.9±2.6	16.1±1.8	9.2±4.8	9.3±2.6	79.1±23.2	4.0±1.6	7.4±3.2	16.5±12.6
4.5 ~ < 5.0	104	111.1±4.6	20.0±2.7	16.2±1.5	8.2±5.0	8.5±1.4	84.9±19.5	4.6±1.9	7.0±3.0	17.5±13.3
5.0 ~ < 5.5	77	114.0±5.1	21.1±3.1	16.2±1.5	7.7±4.6	8.5±1.6	90.6±17.2	4.7±2.0	6.5±2.8	14.4±10.8
5.5 ~ < 6.0	49	116.0±4.3	22.2±3.5	16.5±2.5	6.3±4.8	8.2±1.1	93.7±14.9	4.9±1.7	5.7±1.1	16.2±13.1
6.0 ~ < 6.5	32	117.0±5.6	23.0±4.1	16.7±2.2	7.8±5.1	8.3±1.3	93.2±17.2	5.1±1.8	6.2±2.0	14.6±10.5
6.5 ~ <	18	112.0±5.9	24.9±5.8	17.2±2.7	7.8±5.4	7.9±1.1	95.4±19.8	6.1±2.0	5.7±1.3	14.8±13.6
One-way ANOVA		**	**	ns	**	**	**	**	**	**
**Female (n =436)**										
~ < 3.0	11	97.5±4.1	14.5±1.1$^{\#\#}$	15.3±1.1$^{\#}$	10.5±3.1	11.7±1.8	50.2±14.5	2.0±0.4	9.6±3.7	22.5±14.5
3.0 ~ < 3.5	63	100.5±4.5	15.8±1.7$^{\#\#}$	15.7±1.3$^{\#}$	10.7±3.3$^{\#}$	11.0±2.1	56.9±19.7	2.7±0.9$^{\#\#}$	8.2±3.5	24.6±14.3
3.5 ~ < 4.0	89	106.8±5.8	18.1±2.3	15.8±1.5$^{\#}$	12.0±3.6$^{\#\#}$	9.4±1.6	75.2±17.7	3.7±1.4	7.0±2.3	20.3±14.2
4.0 ~ < 4.5	63	107.6±5.6	17.8±2.3$^{\#\#}$	15.3±1.3$^{\#\#}$	11.7±3.8$^{\#\#}$	9.7±2.0	75.1±16.6	3.5±1.3$^{\#}$	7.1±1.8	18.6±12.5
4.5 ~ < 5.0	60	111.3±5.7	19.7±2.9	15.9±1.5	12.4±4.5$^{\#\#}$	8.8±1.3	78.2±13.5$^{\#}$	4.0±1.3$^{\#}$	6.3±1.5	16.5±13.0
5.0 ~ < 5.5	62	112.8±5.3	20.5±3.1	16.1±1.9	10.7±4.8$^{\#\#}$	8.7±1.2	84.0±16.1$^{\#}$	4.3±1.6	6.3±2.1	15.9±12.5
5.5 ~ < 6.0	47	112.1±4.9$^{\#\#}$	20.6±3.3$^{\#}$	16.4±2.5	11.8±4.7$^{\#\#}$	8.6±1.2	87.7±18.8	4.2±1.3$^{\#}$	6.5±2.0$^{\#}$	15.1±13.3
6.0 ~ < 6.5	18	117.0±5.0	21.6±3.5	15.8±2.4	9.5±4.2	8.2±0.6	85.8±12.1	4.4±0.8	7.2±4.0	15.3±9.4
6.5 ~ <	23	117.8±4.2	23.0±3.9	16.5±2.7	11.3±4.1$^{\#}$	8.2±0.7	87.3±15.3	4.7±1.3$^{\#\#}$	6.2±1.6	14.4±10.9
One-way ANOVA		**	**	*	ns	**	**	**	**	**

$^{*}p < 0.05$, $^{**}P < 0.01$. $^{\#}$Female vs. Male, $p < 0.05$, $^{\#\#}$Female vs. Male, $p < 0.01$.

BMI: body mass index; SRT: shuttle run test; ns: no significance.
